# Antimicrobial Resistance as a Hidden Menace Lurking Behind the COVID-19 Outbreak: The Global Impacts of Too Much Hygiene on AMR

**DOI:** 10.3389/fmicb.2020.590683

**Published:** 2020-12-15

**Authors:** Sama Rezasoltani, Abbas Yadegar, Behzad Hatami, Hamid Asadzadeh Aghdaei, Mohammad Reza Zali

**Affiliations:** ^1^Foodborne and Waterborne Diseases Research Center, Research Institute for Gastroenterology and Liver Diseases, Shahid Beheshti University of Medical Sciences, Tehran, Iran; ^2^Gastroenterology and Liver Diseases Research Center, Research Institute for Gastroenterology and Liver Diseases, Shahid Beheshti University of Medical Sciences, Tehran, Iran; ^3^Basic and Molecular Epidemiology of Gastrointestinal Disorders Research Center, Research Institute for Gastroenterology and Liver Diseases, Shahid Beheshti University of Medical Sciences, Tehran, Iran

**Keywords:** coronavirus disease 2019 (COVID-19), antimicrobial resistance, antibiotics, biocides, disinfectants, selective pressure, mitochondria

## Abstract

The severe acute respiratory syndrome coronavirus 2 (SARS-CoV-2) is a new coronavirus that was recently discovered in 2019. While the world is working hard to overcome and control the coronavirus disease 2019 (COVID-19) pandemic, it is also crucial to be prepared for the great impacts of this outbreak on the development of antimicrobial resistance (AMR). It is predicted that inappropriate and too much use of antibiotics, biocides, and disinfectants during this pandemic may raise disastrous effects on antibiotic stewardship programs and AMR control all around the world. Furthermore, the use of certain antibiotics alone or in combination with antiviral agents or other medications for the treatment of secondary bacterial infections among COVID-19 patients may be regarded as a major factor that negatively affects host immune response by disrupting mitochondrial function and activity. Herein, we suggest that the current management strategies to control AMR and prioritize antibiotic stewardship schemes should be extremely highlighted in relation to the COVID-19 outbreak. The rising concerns about excessive use of antimicrobials and biocides and taking too much hygiene also need to be addressed during this pandemic due to their impacts on AMR, public health, and the environment.

## Introduction

Globally, coronavirus disease 2019 (COVID-19) has created a health catastrophe that has had an immense impact on social care systems, economy, and financial resources. The importance of infection control and prevention measures such as social distancing, hand hygiene, and self-isolation has now been advised at a communal level (Rezasoltani et al., 2020). The COVID-19 pandemic once again emphasizes the necessity to consider bacterial co-infections and secondary infections during and after the viral outbreaks, respectively. Bacterial co-infections have been regarded as an important motive of morbidity and mortality during viral infections and also have been reported to be engaged in COVID-19, although by a low proportion of COVID-19 patients (Huang et al., 2020a; Lansbury et al., 2020; Li et al., 2020). It is noteworthy to indicate that COVID-19 cases that have been administered empiric use of antibiotics may also front onto subsequent complications due to co-infection by antimicrobial-resistant bacterial agents (Rawson et al., 2020). In order to select the appropriate antimicrobials to increase the patient’s survival rate and further inhibit the spread of antimicrobial resistance (AMR), microbiological examination and antibiotic susceptibility testing (AST) of COVID-19 patients presenting with co-infections can be implemented in routine laboratory tests. Moreover, the application of effective narrow-spectrum antibiotics in patients with COVID-19 should be considered wherever possible (Cao et al., 2020). Presently, AMR is responsible for approximately 700,000 deaths every year worldwide (IACG, 2019). It is estimated that due to the current status of the COVID-19 outbreak, AMR will lead to 1,30,000 more deaths by the end of 2020 alone (O’Neill, 2014; Murray, 2020). Unfortunately, it is also predicted that AMR-associated deaths can reach up to 10 million deaths per year by 2050, if the world could not tackle the COVID-19 crisis (O’Neill, 2014).

The World Health Organization (WHO) is worried that the trend will later be fueled by the wrong consumption of antibiotics during the COVID-19 pandemic. Indeed, when physicians are not aware or do not have access to the laboratory diagnostic findings related to the etiology of the infected patients, they tend to drive higher administration of different antimicrobials (Hsu Freelance, 2020). Recent data indicate that only a small proportion of the patients with COVID-19 demands antibiotics to treat further bacterial co-infections (Murray, 2020). Based on a recently issued WHO guideline, antibiotic prophylaxis or treatment of the patients with mild or moderate COVID-19 should be prohibited unless upon a clinical indication (World Health Organization, 2020a). However, great caution should be considered given that inappropriate use or overuse of antibiotics may work as a striking driver of the emergence of AMR (Murray, 2020). Moreover, WHO is extremely concerned due to a significant decline in the investment and lack of innovation for the development of novel antimicrobial agents, which can result in debilitation of global efforts to combat drug-resistant infections (World Health Organization, 2020b).

The uncontrolled use of biocide-based products could lead to the unusual release of such antimicrobials into environmental resources and cause the selective survival of bacterial strains carrying resistance genes during the COVID-19 catastrophe (Buffet-Bataillon et al., 2012; Pal et al., 2015). Furthermore, mitochondria are among the most important intracellular organelles vulnerable to the effects of antibiotic treatment (Tyszka et al., 2020). Since these organelles are in charge of cellular energy production and apoptosis and also are engaged in host immunity against infections (Surbatovic et al., 2013; Singer, 2014), their malfunction could compromise the host’s capability to overcome the COVID-19 infection.

## Antimicrobial Resistance

### Antibiotics

Antimicrobial resistance has been emphasized as the most considerable threat to global health and economy in recent years, but it can hide behind COVID-19 for a while. It is predicted that the current emergence of the COVID-19 pandemic can seriously facilitate the growth rate of AMR worldwide; thus, the continuing and preventive measures against AMR should not be neglected during this global disaster (Lansbury et al., 2020; Murray, 2020). Unfortunately, there were some reports on death of COVID-19 cases who experienced further bacterial co-infections like what was observed for other viral infections (Yap et al., 2004; Centers for Disease Control and Prevention, 2009; Huang et al., 2020b; Li et al., 2020; Wang et al., 2020a). In these patients and in particular upon hospitalization, the situation could be progressively deteriorated due to nosocomial infections by multidrug-resistant (MDR) microorganisms. For instance, during the outbreaks of the former emerging pathogen severe acute respiratory syndrome (SARS) epidemic, there was evidence of an increase in the prevalence of resistant bacterial infections such as methicillin-resistant *Staphylococcus aureus* (MRSA) in the hospital environment (Chai et al., 2005; European Centre for Disease Prevention and Control, 2017). Until now, there are very few data available with respect to cases with COVID-19 contracting bacterial co-infections. It was estimated from separate studies that approximately 1 to 10% of the patients with COVID-19 contracted secondary bacterial co-infections (Lai et al., 2020). In another study in Wuhan (Chen et al., 2020), 11% of death rate was reported among 99 patients with novel coronavirus (2019-nCoV) pneumonia, and most of the cases (71%) were treated with antibiotics; however, only 1% experienced co-infection with bacteria and 4% had fungal co-infections. Moreover, at a single center in Cologne, Germany (Koehler et al., 2020), putative invasive pulmonary aspergillosis (IPA) co-infection was reported in five (26%) of 19 consecutive critically ill COVID-19 patients with moderate to severe acute respiratory distress syndrome (ARDS) without underlying immunocompromising disease who were admitted to two separate intensive care units (ICUs). Also, Heard et al. (2020) suggested that COVID-19 fungal research should consider invasive *Candida* spp. as a potential COVID-19-associated superinfection and the potential harm of antifungal therapy in the absence of diagnostically confirmed COVID-19-associated pulmonary aspergillosis (CAPA). Based on their approach, empirical antifungal therapy does not seem to be beneficial, and about 21% of patients who received empirical antifungals such as voriconazole, isavuconazole, and caspofungin had adverse effects without providing a survival benefit. Therefore, incorporation of strategic algorithms for accurate diagnosis and effective treatment of invasive fungal disease is vital in critically ill patients with COVID-19 and subsequently may result in a definitive diagnosis and successful antifungal therapy throughout the period of severe respiratory distress.

Additionally, in a single-center study in Wuhan that involved 36 non-survivors with COVID-19, antibiotic therapy was administered to all cases, and 61.1% used combination therapy and 38.9% were on a single antibiotic consumption (Huang et al., 2020b). Also, based on a report from the *Istituto Superiore di Sanità* in Italy, 84% of the expired SARS coronavirus 2 (SARS-CoV-2)-positive patients used antibiotic therapy, whereas 54% received antiviral therapy and 31% received steroid therapy during hospitalization (Istituto Superiore di Sanità, 2020). It should be noted that there was no information on microbial detection in the two former studies. Compared to the pandemic H1N1 influenza, which was accompanied by 12–19% of secondary bacterial infections, SARS-CoV-2 resulted in up to 10% bacterial co-infections (Kim, 2020). Although antibiotic therapy to combat bacterial pneumonia is generally accepted in clinical practice for H1N1 influenza, the condition is essentially different and unclear in SARS-CoV-2-related pneumonia. On the other hand, antibiotic or antiviral therapy to treat co-infections in patients with COVID-19 could be regarded as an effective approach, although these patients are in minority (No Authors, 2020). For instance, teicoplanin, a narrow-spectrum antibiotic that is primarily active against Gram-positive organisms such as staphylococcal infections, previously exhibited efficacy to hinder the first stage of the viral life cycle of the Middle East respiratory syndrome coronavirus (MERS-CoV) in host cells (Zhou et al., 2016). Recently, it was proposed that this antibiotic can be potentially recommended as a promising treatment option for patients with SARS-CoV-2 (formerly 2019-nCoV) (Baron et al., 2020). Despite low reports of laboratory-confirmed secondary bacterial co-infections, there have been comparatively a high level (approximately up to 45% of the COVID-19 patients received antibiotics) of antibiotic consumption during COVID-19 treatment (Lai et al., 2020; Xu et al., 2020). Furthermore, it should be noted that differentiating COVID-19 infection from bacterial pneumonia seems to be problematic mainly due to unneeded antibiotic usage for several cases without confirmed secondary bacterial co-infections (Huttner et al., 2020).

Apparently, antibiotic prophylaxis is mostly applied to prevent further bacterial co-infections among the hospitalized cases as seen for the patients with COVID-19 (Huang et al., 2020a; Lupia et al., 2020; Wang et al., 2020b; World Health Organization, 2020a). It is noteworthy to be highlighted that the inappropriate use of antibiotics could considerably and silently lead to AMR development during this global outbreak (Murray, 2020). Unfortunately, recent studies reveal that, in several countries, common and extensive use of antibiotic treatment for COVID-19 hospitalized patients is considered as a part of the routine treatment package (No Authors, 2020). For instance, in 191 hospitalized COVID-19 patients in Wuhan, 95% of them received antibiotic therapies and 21% received antiviral treatments (Zhou et al., 2020). However, these practices significantly differ around various clinical settings worldwide. In a report from the United States, out of 393 COVID-19 cases in New York, only 5.6% of the patients had bacteremia, and surprisingly, none of them received antibiotic therapy (Goyal et al., 2020). Therefore, taking coordinated interventions and performing antibiotic stewardship programs (ASPs) are strongly recommended to limit antibiotic consumptions and prevent the emergence and transmission of antimicrobial−resistant bacteria during the COVID-19 pandemic (No Authors, 2020). Moreover, the collection of valid data and records on the prevalence of AMR before and after the pandemic would be helpful to evaluate the efficacy of these interventions and preventive measures. Also, microbial genomic comparative analysis of the DNA sequences involved in the drug resistance before, during, and after the COVID-19 pandemic could provide useful information on the rate of genetic alterations and for clarifying the potential mechanisms behind the AMR development (Ruan and Feng, 2016).

### Biocides and Disinfectants

**FIGURE 1:**
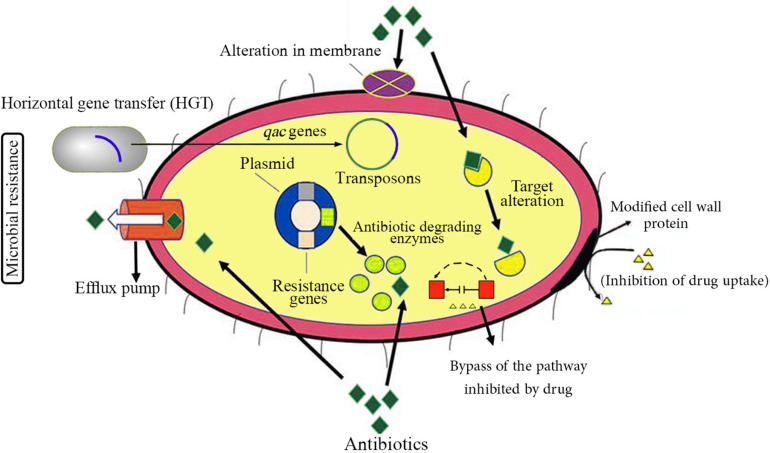
Schematic representation of different antibiotic resistance mechanisms in bacteria. Apparently, most pathogenic bacterial species have the potential capability of developing resistance to at least some antimicrobial agents. There are two types of antibiotic resistance: intrinsic (or inherent) resistance and acquired resistance. Intrinsic antibiotic mechanisms are normally chromosome-encoded; on the other hand, acquired resistance mechanisms are generally obtained by horizontal gene transfer (HGT) and include plasmid-encoded and transposons-mediated antibiotic resistance. The main mechanisms of antibiotic resistance are alteration in membrane components leading to reduced permeability of the cell membrane, modification in cell wall proteins as the common antibiotic targets, inhibiting or limiting uptake of a drug, bypassing the pathway (compensatory tack) inhibited by a drug, degrading and inactivation of a drug by modification/degradation enzymes, and pumping out of a drug by various types of active efflux pumps.

**FIGURE 2:**
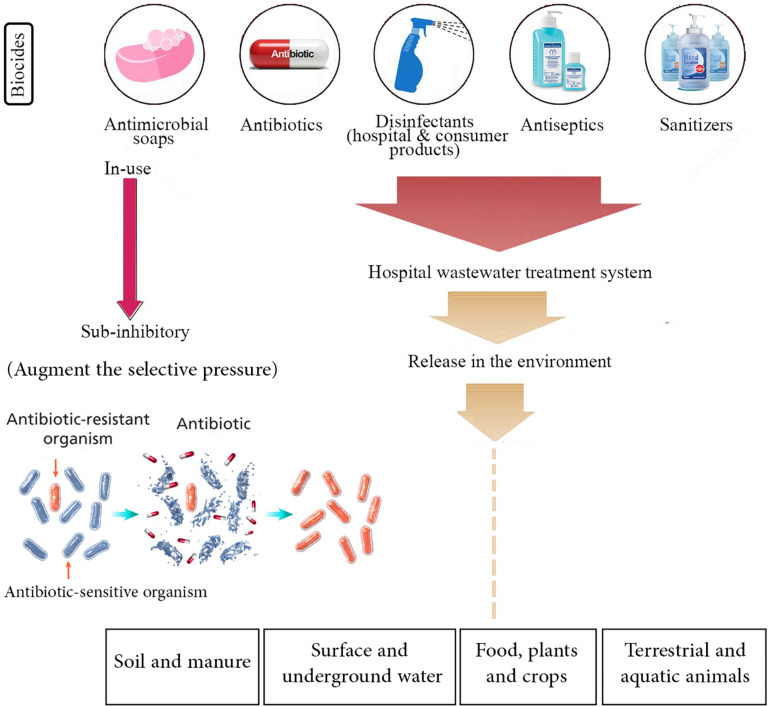
Negative impact of high-concentration release of different kinds of biocides and antibiotics in the environment, which may result in antibiotic resistance enrichment and resistance dissemination. It is presumed that during the COVID-19 pandemic, these antimicrobials are excessively released in the soil and manure, surface and underground water, food, plants and crops, and terrestrial and aquatic animals. On the other hand, enrichment of biocide and antibiotic concentrations at the sub-minimum inhibitory concentration (sub-MIC) in the environment may augment the selective pressure phenomenon, boost the horizontal gene transfer (HGT), and drive the evolution of antimicrobial resistance (AMR) that lead to the selection of antibiotic-resistant bacteria. Therefore, current management practices and strategies to prevent and control AMR should be extremely highlighted in relation to the COVID-19 outbreak.

Since the beginning of the COVID-19 outbreak, globally, there have been huge efforts to prevent further transmission of the virus by applying social distancing inside the engaged countries and closing international borders. Similar to previous respiratory outbreaks, WHO strongly recommended to simply and regularly wash hands with soap and water or use alcohol-based hand sanitizer and other disinfectants to kill the viruses. It seems that the gigantic use of disinfectants may be implemented for a prolonged time as long as the pandemic remains all over the world. Although these hygiene practices may decline the dissemination of AMR that is regarded as a promising consequence, there is also a possibly threatening impact for emerging drug resistance, which can originate from elevated consumption of such antimicrobial products (Buffet-Bataillon et al., 2012; Murray, 2020). The antimicrobial activity of most of these products is basically due to the presence of biocide materials in their formulations. Generally, biocides are antimicrobial compounds present in household disinfectants, sanitizers, and cleaners, whose inappropriate usage might consequently result in the occurrence of AMR (Levy, 2002; Pal et al., 2015; Webber et al., 2015). Figure 1 schematically represents different antibiotic resistance mechanisms exploited by bacteria. Besides, there is much environmental concern about the unusual release and dissemination of higher concentrations of biocide-based products into the surface and underground waters and also wastewater treatment systems during the SARS-CoV-2 pandemic (Buffet-Bataillon et al., 2012). The negative impact of high-concentration release of different kinds of biocides and antibiotics in the environment and their effects on AMR enrichment and resistance dissemination are demonstrated in Figure 2.

It should be noted that the final concentration of disinfectants and biocides released in the environment such as soil, water, ponds, and more importantly in the microecosystems (MESs) and microecological niches where various bacterial species are present is critical. Generally, it is supposed that high concentrations of such microbicidal agents could completely kill most bacterial species especially those that provide beneficial services to the ecosystem and to other living organisms while inhibiting the emergence of drug-resistant microorganisms (Murray, 2020). On the other hand, it is also presumed that if the biocide concentrations reach the sub-minimum inhibitory concentration (sub-MIC), this event may augment the selective pressure, boost the horizontal gene transfer (HGT), and drive the evolution of AMR (McBain et al., 2002; Gilbert and McBain, 2003). For instance, these events may lead to increased efflux-mediated mechanisms of resistance, both agent-specific and multidrug, which are regarded as significant determinants of intrinsic and/or acquired resistance to the antibiotics, and also reduced biocide susceptibility following mutational hyperexpression of the efflux pumps encoding genes (Poole, 2005; Maillard, 2007). Furthermore, we hypothesize that a higher rate of biocide exposure through different routes including inhalation, oral, dermal contact, as well as eye contact can occur during the COVID-19 pandemic by spray application or when biocides evaporate from products and treated articles. As mentioned above, based on laboratory experiments, biocides may result in an elevated selective pressure toward antibiotic resistance and other disinfectants. According to Randall et al. (2004), *Salmonella enterica*, a common foodborne pathogen that is associated with salmonellosis, was capable of tolerating relatively high concentrations of phenolic disinfectant or triclosan and of developing cross-resistance to certain antibiotics mostly *via* increased frequency of mutation, approximately 10- to 100-fold following exposure to the biocides. Current data also suggest that some types of disinfectants can be taken *via* inhalation route and oral pathways and, after crossing the biological barriers, can accumulate in host tissues including lungs, liver, kidneys, stomach, brain, and blood, resulting in adverse effects on human health (Li et al., 2018).

It should be noted that understanding the transferability of disinfectant resistance genes can considerably help to limit the outspread of such mobile genetic elements among microorganisms (Kampf, 2018; Donaghy et al., 2019). Albeit the precise mechanisms of gene transfer of disinfectant-associated resistance genes still remain unrecognized, a few mechanisms have been suggested (Ramsay et al., 2016; Partridge et al., 2018; McCarlie et al., 2020). For example, certain conjugative plasmids have been detected to carry disinfectant and antibiotic resistance genes (ARGs) such as *qac* genes in *Staphylococcus*, *Enterococcus*, and *Escherichia coli* (Jia et al., 2008; Braga et al., 2011; Wassenaar et al., 2015). Taken together, a better understanding of resistance mechanisms and gene transfer of putative genes involved in antibiotic and biocide resistance could help prevent the emergence and evolution of AMR in the current pandemic.

### Antibiotics and Mitochondria

Since there is currently no specific and effective therapeutic agent, medication, or vaccines available to treat COVID-19 patients, the human immune system is considered as the solely active weapon against the virus. Unfortunately, off-target sites of majority of the approved antibiotics include mitochondria, which are evolutionary linked to prokaryote cells (Tyszka et al., 2020). Mitochondria are membrane-bound organelles present in almost all eukaryotic cells and responsible for orchestrating cellular energy production and acting as signaling hubs in antibacterial and antiviral immune responses (Osellame et al., 2012). Thus, antibiotic therapy could cause a malfunction of mitochondrial physiology, which in turn may weaken the host immune response against the COVID-19 infection. A number of antibiotics consisting of such applied to COVID-19 in China and other countries have been reported to inhibit mitochondrial activity, DNA synthesis, and biogenesis (Tyszka et al., 2020). These antimicrobial agents can affect mitochondrial function and ATP production and also induce cell death pathways (Surbatovic et al., 2013; Singer, 2014; Zhang et al., 2020). For example, azithromycin that has been administered to treat patients with COVID-19 could cause mitochondrial toxicity, overproduction of reactive oxygen species (ROS), and DNA oxidative damage (Tyszka et al., 2020). However, the present data suggest that co-administration of azithromycin with hydroxychloroquine showed promising outcomes in a small proportion of COVID-19 patients and could reduce or eliminate the viral load (Gautret et al., 2020). This once again confirms appropriate selection of the dedicated antibiotics against COVID-19 infection, although their risk evaluation should be considered (Murray, 2020). Presently, there is limited information about the effectiveness of macrolides alone or combined with hydroxychloroquine for the treatment of COVID-19 patients. Moreover, both macrolide antibiotics and hydroxychloroquine can increase the QT interval; thus, this drug combination may result in cardiovascular harms (The Center for Evidence Based Medicine, 2020). It is noteworthy to mention that the grade of sensitivity to side effects of antibiotics can differ from one person to another. Importantly, it is proposed that patients with underlying mitochondrial disorders are much more sensitive to certain antibiotics than the normal population (Stoker et al., 2019). However, there is much controversy whether antibiotics favor current treatments used for COVID-19 or may interfere with the host immune functions. Further investigations and clinical trials are urgently needed to answer this question.

## Concluding Remarks

Before the emergence of the SARS-CoV-2 pandemic, the world was already demanding immediate, coordinated, and ambitious actions to avert the potentially disastrous AMR crisis and its related economic and health consequences. Now that we are in the midst of a global crisis, which is disrupting normal life for everyone, causing hundreds of thousands of people sick or hospitalized, and killing some of the most high-risk individuals, a better understanding of the pathophysiology of COVID-19 and advocating of potential effective therapeutics for the treatment of secondary bacterial co-infections seem to be critical. Also, prospective monitoring of the co-infections in patients with COVID-19 could help apprehend whether such co-infections affect disease progression and may interfere with antiviral treatment. Furthermore, current management practices and strategies to control AMR and prioritize antibiotic stewardship schemes should be extremely highlighted in relation to the COVID-19 outbreak. The concerns about consumption of “too many antimicrobials and biocides” and taking “too much hygiene” also need to be addressed during this pandemic due to their impacts on AMR, public health, and the environment.

## Author Contributions

SR and AY contributed significantly to the literature review and wrote the draft of the manuscript. AY worked on concept and design of the study and interpreted the collected information. BH, HA, and MZ provided clinical advice and guidance for the improvement of the manuscript. AY critically revised the final version of the manuscript. All authors approved the final version of the manuscript and the authorship list.

## Conflict of Interest

The authors declare that the research was conducted in the absence of any commercial or financial relationships that could be construed as a potential conflict of interest.

## References

[B1] Baron S. A. Devaux C. Colson P. Raoult D. Rolain J. M. (2020). Teicoplanin: an alternative drug for the treatment of coronavirus COVID-19? *Int. J. Antimicrob. Agents.* 55:105944 10.1016/j.ijantimicag.2020.105944 PMC7102624 32179150

[B2] Braga T. Marujo P. Pomba C. Lopes M. (2011). Involvement and dissemination of the enterococcal small multidrug resistance transporter QacZ in resistance to quaternary ammonium compounds. *J. Antimicrob. Chemother.* 66:2. 10.1093/jac/dkq460 21147826

[B3] Buffet-Bataillon S. Tattevin P. Bonnaure-Mallet M. Jolivet-Gougeon A. (2012). Emergence of resistance to antibacterial agents: the role of quaternary ammonium compounds-a critical review. *Int. J. Antimicrob. Agents.* 39:5. 10.1016/j.ijantimicag.2012.01.011 22421329

[B4] Cao J. Tu W. Cheng W. Yu L. Liu Y. Hu X. (2020). Clinical features and short-term outcomes of 102 patients with corona virus disease 2019 in Wuhan, China. *Clin. Infect. Dis.* 71:15 10.1093/cid/ciaa243 PMC7184479 32239127

[B5] Centers for Disease Control and Prevention (2009). Bacterial Coinfections in Lung Tissue Specimens from Fatal Cases of 2009 Pandemic influenza A (H1N1)-United States, May-August 2009. MMWR Morb Mortal Wkly Rep. 58:38. Available online at: https://www.cdc.gov/mmwr/preview/mmwrhtml/mm58e0929a1.htm (accessed September 29, 2009). 19798021

[B6] Chai L. Y. A. Ng T. M. Habib A. G. Singh K. Kumarasinghe G. Tambyah P. A. (2005). Paradoxical increase in methicillin-resistant *Staphylococcus aureus* acquisition rates despite barrier precautions and increased hand washing compliance during an outbreak of severe acute respiratory syndrome. *Clin. Infect. Dis.* 40:4. 10.1086/427150 15712092 PMC7107850

[B7] Chen N. Zhou M. Dong X. Qu J. Gong F. Han Y. (2020). Epidemiological and clinical characteristics of 99 cases of 2019 novel coronavirus pneumonia in Wuhan, China: a descriptive study. *Lancet* 395:10223. 10.1016/S0140-6736(20)30211-7 PMC7135076 32007143

[B8] Donaghy J. A. Jagadeesan B. Goodburn K. Grunwald L. Jensen O. N. Jespers A. D. (2019). Relationship of sanitizers, disinfectants, and cleaning agents with antimicrobial resistance. *J. Food Prot.* 82:5. 10.4315/0362-028X.JFP-18-373 31021666

[B9] European Centre for Disease Prevention and Control (2017). *ECDC Country Visit to Italy to Discuss Antimicrobial Resistance Issues.* Available online at: https://www.ecdc.europa.eu/en/publications-data/ecdc-country-visit-italy-discuss-antimicrobial-resistance-issues

[B10] Gautret P. Lagier J. Parola P. Hoang V. Meddeb L. Mailhe M. (2020). Hydroxychloroquine and azithromycin as a treatment of COVID-19: results of an open label non-randomized clinical trial. *Int. J. Antimicrob. Agents.* 56:1. 10.1016/j.ijantimicag.2020.105949 32205204 PMC7102549

[B11] Gilbert P. McBain A. J. (2003). Potential impact of increased use of biocides in consumer products on prevalence of antibiotic resistance. *Clin. Microbiol. Rev.* 16:2. 10.1128/CMR.16.2.189-208.2003 12692093 PMC153147

[B12] Goyal P. Choi J. J. Pinheiro L. C. Schenck E. J. Chen R. Jabri A. (2020). Clinical characteristics of covid-19 in New York city. *N. Engl. J. Med.* 382:24. 10.1056/NEJMc2010419 PMC7182018 32302078

[B13] Heard K. L. Hughes S. Mughal N. Moorea L. S. P. (2020). COVID-19 and fungal superinfection. *Lancet Microbe*. 1:e107 10.1016/S2666-5247(20)30065-3 PMC7333994 32835341

[B14] Hsu Freelance J. (2020). How covid-19 is accelerating the threat of antimicrobial resistance. *BMJ* 369:m1983. 10.1136/bmj.m1983 32423901

[B15] Huang C. Wang Y. Li X. Ren L. Zhao J. Hu Y. (2020a). Clinical features of patients infected with 2019 novel coronavirus in Wuhan, China. *Lancet.* 395:10223 10.1016/S0140-6736(20)30183-5 PMC7159299 31986264

[B16] Huang Y. Yang R. Xu Y. Gong P. (2020b). Clinical characteristics of 36 non-survivors with COVID-19 in Wuhan, China. *medRxiv* [Preprint]. 10.1101/2020.02.27.20029009

[B17] Huttner B. D. Catho G. Pano-Pardo J. R. Pulcini C. Schouten J. (2020). COVID-19: don’t neglect antimicrobial stewardship principles! *Clin. Microb. Infect.* 26:7. 10.1016/j.cmi.2020.04.024 32360446 PMC7190532

[B18] IACG (2019). *No Time to Wait: Securing the Future from Drug-Resistant Infections.* Available online at: https://www.who.int/antimicrobial-resistance/interagency-coordination-group/final-report/en

[B19] Istituto Superiore di Sanità (2020). *Characteristics of COVID-19 patients dying in Italy.* Available online at: https://www.epicentro.iss.it/en/coronavirus/sars-cov-2-analysis-of-deaths (accessed November 18, 2009).

[B20] Jia B. Zhou T. Huang A. Huang W. X. (2008). Role of TMS5: Staphylococcal multidrug-efflux protein QacA. *Chin. Med. J.* 121 409–413. 10.1097/00029330-200803010-00008 18364112

[B21] Kampf G. (2018). Biocidal agents used for disinfection can enhance antibiotic resistance in gram-negative species. *Antibiotics (Basel).* 7:110. 10.3390/antibiotics7040110 30558235 PMC6316403

[B22] Kim H. (2020). Outbreak of novel coronavirus (COVID-19): what is the role of radiologists? *Eur. Radiol.* 18 1–2. 10.1007/s00330-020-06748-6742 PMC7087878 32072255

[B23] Koehler P. Cornely O. Böttiger B. Dusse F. Eichenauer D. Frieder Fuchs F. (2020). COVID-19 associated pulmonary aspergillosis. *Mycoses*. 63 528–534. 10.1111/myc.13096 32339350 PMC7267243

[B24] Lai C. C. Shih T. P. Ko W. C. Tang H. J. Hsueh P. R. (2020). Severe acute respiratory syndrome coronavirus 2 (SARS-CoV-2) and coronavirus disease-2019 (COVID-19): The epidemic and the challenges. *Int. J. Antimicrobial. Agents.* 55:105924. 10.1016/j.ijantimicag.2020.105924 32081636 PMC7127800

[B25] Lansbury L. Lim B. Baskaran V. Shen L. W. (2020). Co-infections in people with COVID-19: a systematic review and meta-analysis. *J. Infect.* 81:46. 10.1016/j.jinf.2020.05.046 32473235 PMC7255350

[B26] Levy S. B. (2002). Active efflux, a common mechanism for biocide and antibiotic resistance. *J. Appl. Microbiol.* 92 655–715. 10.1046/j.1365-2672.92.5s1.4.x 12000614

[B27] Li W. Calle L. M. Hanford A. J. Stambaugh I. Callahan M. R. (2018). “Investigation of silver biocide as a disinfection technology for spacecraft-an early literature review,” in *Proceedings of the 48th International Conference on Environmental Systems ICES-2018-82*, Albuquerque, NM.

[B28] Li X. Wang L. Yan S. Yang F. Xiang L. Zhu J. (2020). Clinical characteristics of 25 death cases with COVID-19: a retrospective review of medical records in a single medical center, Wuhan, China. *Int. J. Infect. Dis.* 94 128–132. 10.1016/j.ijid.2020.03.053 32251805 PMC7128884

[B29] Lupia T. Scabini S. Pinna S. M. Perri G. D. De Rosa F. G. Corcione S. (2020). 2019 novel coronavirus (2019-nCoV) outbreak: a new challenge. *J. Glob. Antimicrob. Resist.* 21 22–27. 10.1016/j.jgar.2020.02.021 32156648 PMC7102618

[B30] Maillard J. (2007). Bacterial resistance to biocides in the healthcare environment: should it be of genuine concern? *J. Hosp. Infect.* 65 60–72. 10.1016/S0195-6701(07)60018-8 17540245

[B31] McBain A. J. Rickard A. H. Gilbert P. (2002). Possible implications of biocide accumulation in the environment on the prevalence of bacterial antibiotic resistance. *J. Ind. Microbiol. Biotechnol.* 29:6. 10.1038/sj.jim.7000324 12483474

[B32] McCarlie S. Boucher C. E. Bragg R. (2020). Molecular basis of bacterial disinfectant resistance. *Drug Resist. Updat.* 48:100672. 10.1016/j.drup.2019.100672 31830738

[B33] Murray A. K. (2020). The novel coronavirus covid-19 outbreak: global implications for antimicrobial resistance. *Front. Microbiol.* 11:1020. 10.3389/fmicb.2020.01020 32574253 PMC7237633

[B34] No Authors (2020). Antimicrobial resistance in the age of COVID-19. *Nat. Microbiol.* 5:779. 10.1038/s41564-020-0739-4 32433531

[B35] O’Neill J. (2014). *Antimicrobial Resistance: Tackling a Crisis for the Health and Wealth of Nations.* O’Neill Report Wellcome Trust. Review on Antimicrobial Resistance. (O’Neill). Available online at: https://wellcomecollection.org/works/rdpck35v

[B36] Osellame L. D. Blacker T. S. Duchen M. R. (2012). Cellular and molecular mechanisms of mitochondrial function. *Best Pract. Res. Clin. Endocrinol. Metab.* 26:6. 10.1016/j.beem.2012.05.003 23168274 PMC3513836

[B37] Pal C. Bengtsson-Palme J. Kristiansson E. Larsson D. G. (2015). Cooccurrence of resistance genes to antibiotics, biocides and metals reveals novel insights into their co-selection potential. *BMC Genomics.* 16:964. 10.1186/s12864-015-2153-5 26576951 PMC4650350

[B38] Partridge S. R. Kwong S. M. Firth N. Jensen S. O. (2018). Mobile genetic elements associated with antimicrobial resistance. *Clin. Microbiol. Rev.* 31:4. 10.1128/CMR.00088-17 30068738 PMC6148190

[B39] Poole K. (2005). Efflux-mediated antimicrobial resistance. *J. Antimicrob. Chemother.* 56:1. 10.1093/jac/dki171 15914491

[B40] Ramsay J. P. Kwong S. M. Murphy R. J. T. Eto K. Y. Price K. J. Nguyen Q. T. (2016). An updated view of plasmid conjugation and mobilization in Staphylococcus. *Mob. Genet. Elements*. 6;4. 10.1080/2159256X.2016.1208317 27583185 PMC4993578

[B41] Randall L. P. Cooles S. W. Piddock L. J. V. Woodward M. J. (2004). Effect of triclosan or a phenolic farm disinfectant on the selection of antibiotic-resistant *Salmonella enterica*. *J. Antimicrob. Chemother.* 54:3. 10.1093/jac/dkh376 15269199

[B42] Rawson T. M. Ming D. Ahmad R. Moore L. S. P. Holmes A. H. (2020). Antimicrobial use, drug-resistant infections and COVID-19. *Nat. Rev. Microbiol.* 18 409–410. 10.1038/s41579-020-0395-y 32488173 PMC7264971

[B43] Rezasoltani S. Hatami B. Yadegar A. Asadzadeh Aghdaei H. Zali M. R. (2020). How patients with chronic liver diseases succeed to deal with COVID-19? *Front. Med.* 7:398. 10.3389/fmed.2020.00398 32754608 PMC7381291

[B44] Ruan Z. Feng Y. (2016). BacWGSTdb, a database for genotyping and source tracking bacterial pathogens. *Nucleic Acids Res.* 44 D682–D687. 10.1093/nar/gkv1004 26433226 PMC4702769

[B45] Singer M. (2014). The role of mitochondrial dysfunction in sepsis-induced multi-organ failure. *Virulence.* 5:1. 10.4161/viru.26907 24185508 PMC3916385

[B46] Stoker M. L. Newport E. Hulit J. C. West A. P. Morten K. J. (2019). Impact of pharmacological agents on mitochondrial function: a growing opportunity? *Biochem. Soc. Trans.* 47:6. 10.1042/BST20190280 31696924 PMC6925523

[B47] Surbatovic M. Jevdjic J. Veljovic M. Popovic N. Djordjevic D. Radakovic S. (2013). Immune response in severe infection: could life-saving drugs be potentially harmful? *Sci. World J.* 2013;961852. 10.1155/2013/961852 24198733 PMC3806431

[B48] The Center for Evidence Based Medicine (2020). *The Center for Evidence Based Medicine Develops, Promotes and Disseminates Better for Health Are. What is the Evidence for Using Macrolide Antibiotics to Treat COVID-19?.* Available online at: https://www.cebm.net/covid-19/what-is-the-evidence-for-use-of-macrolide-antobiotics-for-treatmetnof-covid-19 (accessed April 28, 2020).

[B49] Tyszka J. Kobos K. Tyszka A. (2020). Antibiotics against COVID-19 and mitochondria? Urgent thinking out of the box. [Preprints]. 10.20944/preprints202004.0269.v1 32283112

[B50] Wang L. He W. Yu X. Hu D. Bao M. Liu H. (2020a). Coronavirus Disease 2019 in elderly patients: characteristics and prognostic factors based on 4-week follow-up. *J. Infect.* 80:6. 10.1016/j.jinf.2020.03.019 32240670 PMC7118526

[B51] Wang Z. Yang B. Li Q. Wen L. Zhang R. (2020b). Clinical features of 69 cases with coronavirus disease 2019 in Wuhan, China. *Clin. Infect. Dis.* 71:15. 10.1093/cid/ciaa272 32176772 PMC7184452

[B52] Wassenaar T. Ussery D. Nielsen L. Ingmer H. (2015). Review and phylogenetic analysis of qac genes that reduce susceptibility to quaternary ammonium compounds in *Staphylococcus* species. *Eur. J. Microbiol. Immunol.* 5:1. 10.1556/EuJMI-D-14-00038 25883793 PMC4397847

[B53] Webber M. A. Whitehead R. N. Mount M. Loman N. J. Pallen M. J. Piddock L. J. (2015). Parallel evolutionary pathways to antibiotic resistance selected by biocide exposure. *J. Antimicrob. Chemother.* 70:8. 10.1093/jac/dkv109 25953808 PMC4500774

[B54] World Health Organization (2020a). *Record Number of Countries Contribute Data Revealing Disturbing Rates of Antimicrobial Resistance.* Available online at: https://www.who.int/news-room/detail/01-06-2020-record-number-of-countries-contribute-data-revealing-disturbing-rates-of-antimicrobial-resistance (accessed June 1, 2020).

[B55] World Health Organization (2020b). *Strategic Preparedness and Response Plan for the New Coronavirus.* Available online at: https://www.who.int/publications-detail/covid-19-strategy-update-13-april-2020 (accessed April 15, 2020).

[B56] Xu X. W. Wu X. X. Jiang X. G. Xu K. J. Ying L. J. Ma C. L. (2020). Clinical findings in a group of patients infected with the 2019 novel coronavirus (SARS-Cov-2) outside of Wuhan, China: retrospective case series. *BMJ* 368:m606. 10.1136/bmj.m606 32075786 PMC7224340

[B57] Yap F. H. Y. Gomersall C. D. Fung K. S. C. Ho P. L. Ho O. M. Lam P. K. N. (2004). Increase in methicillin-resistant *Staphylococcus aureus* acquisition rate and change in pathogen pattern associated with an outbreak of severe acute respiratory syndrome. *Clin. Infect. Dis*. 39:4. 10.1086/422641 15356814 PMC7204093

[B58] Zhang L. Huang B. Xia H. Fan H. Zhu M. Zhu L. (2020). Retrospective analysis of clinical features in 134 coronavirus disease 2019 cases. *Epidemiol. Infect*. 148:e199 10.1017/S0950268820002010 PMC7487751 32878654

[B59] Zhou F. Yu T. Du R. Fan G. Liu Y. Liu Z. (2020). Clinical course and risk factors for mortality of adult in patients with COVID-19 in Wuhan, China: a retrospective cohort study. *Lancet.* 395:10229 10.1016/S0140-6736(20)30566-3 PMC7270627 32171076

[B60] Zhou N. Pan T. Zhang J. Li Q. Zhang X. Bai C. (2016). Glycopeptide antibiotics potently inhibit cathepsin l in the late endosome/lysosome and block the entry of ebola virus, Middle East respiratory syndrome coronavirus (MERS-CoV) and severe acute respiratory syndrome coronavirus (SARS-CoV). *J. Biol. Chem.* 291:17. 10.1074/jbc.M116.716100 26953343 PMC4861487

